# Real-time shear wave elastography (SWE) assessment of short- and long-term treatment outcome in Budd-Chiari syndrome: A pilot study

**DOI:** 10.1371/journal.pone.0197550

**Published:** 2018-05-30

**Authors:** Hong-Wei Wang, Hua-Ning Shi, Jia Cheng, Fang Xie, Yu-Kun Luo, Jie Tang

**Affiliations:** 1 Department of Ultrasound, Chinese PLA General Hospital, Beijing, China; 2 Department of Ultrasound, Yong Liang Hospital, Baoding, China; 3 Department of Ultrasound, Affiliated Hospital of Chengde Medical College, Chengde, China; Medizinische Fakultat der RWTH Aachen, GERMANY

## Abstract

**Purpose:**

The first aim of this study was to analyze the relationships between liver stiffness measurement, hepatic venous pressure and liver fibrosis. The second aim was to demonstrate the utility of real-time shear wave elastography for evaluation of Budd-Chiari syndrome patients before and after balloon hepatic venous angioplasty.

**Materials and methods:**

A total of 32 patients with Budd-Chiari syndrome slated for successful balloon angioplasty met the inclusion and exclusion criteria. Shear wave elastography was used to generate dynamic liver stiffness measurement 2 days before angioplasty and 2 days, 3 months, and 6 months after angioplasty. Hepatic venous pressures were measured during balloon angioplasty. Correlations among liver stiffness, hepatic venous pressure, and fibrosis were assessed.

**Result:**

Mean liver stiffness was 35.17 ± 10.60 kPa, 20.15 ± 5.47 kPa, 15.36 ± 4.34 kPa and 15.68 ± 5.58 kPa at baseline and 2 days, 3 months, and 6 months after angioplasty, respectively. Liver stiffness measured at 2 days and 3 months after angioplasty was significantly decreased (P < 0.001); liver stiffness measured at 6 months after angioplasty was not significantly different from that measured at 3 months after angioplasty (P = 0.636). Analysis of liver stiffness measurement and hepatic venous pressure before balloon angioplasty yielded a coefficient of correlation r = 0.701 (P < 0.001). Before and 2d after angioplasty, liver stiffness measurement did not correlated with fibrosis (r = − 0.170, P = 0.22), (r = 0.223, P = 0.220), respectively, while the LSM difference before and 2 days after angioplasty negatively correlated with stiffness severity (r = − 0.502, P = 0.003). Liver stiffness measured at 2 days and 3 months after angioplasty was significantly decreased (P < 0.001), remaining stable at 3 months, though still in the cirrhotic range.

**Conclusions:**

The liver stiffness of Budd-Chiari syndrome patients, measured by shear wave elastography, decreased considerably after hepatic venous recanalization, and significantly correlated with hepatic venous pressure though not with degree of fibrosis. Shear wave elastography may be effective in monitoring short- and long-term treatment outcomes in Budd-Chiari syndrome.

## Introduction

Elastography uses ultrasound to estimate the stiffness of a material when a mechanical stress is applied. Shear wave elastography (quantitative elastography), such as transient elastography (TE), real-time shear wave elastography (SWE), and point shear-wave elastography (pSWE), is a noninvasive methodology that has been used to monitor liver stiffness in patients with chronic viral hepatitis [1–3]. Transient elastography (Fibroscan; Echosense, Paris, France) is the earliest and the most validated elastography imaging technique for liver stiffness [4]. The European Association for the Study of the Liver (EASL) guidelines for patients with chronic hepatitis B and C [4,5] recommend TE as an alternative to liver biopsy to assess liver stiffness owing to its accuracy, non-invasiveness and easy acceptance among patients. However, it is impossible to obtain results from patients with ascites, and its use with a one-dimensional ultrasound system has certain limitations [5].

SWE is the most recently developed technique [6]. Like TE, SWE calculates the speed of a shear wave to provide a quantitative estimate of tissue stiffness. Unlike TE, SWE makes breakthroughs in the following aspects [7]: the equipment is incorporated into the ultrasound system with the use of an ultrafast, ultrasonic scanner and the final display includes both color-coded images and numeric values. It can also be used in the presence of ascites. Studies of the use of SWE in liver stiffness measurement (LSM) and correlations between liver stiffness and fibrosis [8,9] have suggested a stronger and more accurate correlation between LSM and fibrosis with SWE compared with TE [10,11].

However, recent studies have found that LSM is not only affected by the fibrosis stage but also by other confounding factors, such as the levels of aminotransferases [12,13], extrahepatic cholestasis [14], food intake [15–17], breath holding [18], and intake of beta-blockers [19]. In addition, previous studies have found that hepatic congestion can lead to an increase in LSM [20–22], but patients’ hepatic venous pressures were not measured, and the relationships among LSM, hepatic venous pressure and liver fibrosis were not analyzed.

We collected Budd-Chiari syndrome (BCS) patients who have undergone balloon angioplasty. BCS is defined as any level of hepatic venous outflow obstruction from the junction of small vein to the inferior vena cava and the right atrium, regardless of the cause of obstruction [23]. This leads to an increase in hepatic vein pressure, and, if prolonged, it will result in fibrosis [23]. For patients with short-segment stenosis, balloon angioplasty with or without stenting is considered ideal to restore venous outflow in the stenotic hepatic vein or inferior vena cava (IVC) [24, 25]. Hepatic venous pressure (cmH_2_O) can be measured during balloon angioplasty, and a decline in hepatic venous pressure is considered to be reliable sign of successful vessel dilatation [23].

Therefore, the first aim of this study was to analyze the relationships among LSM, hepatic venous pressure and liver fibrosis. The second aim was to evaluate the usefulness of SWE for evaluation of patients with BCS before and after balloon angioplasty.

## Materials and methods

### Ethical statement

This prospective single-institution study was performed in strict accordance with the ethical guidelines of the Declaration of Helsinki, and was approved by the Ethics Committee of Chinese People's Liberation Army General Hospital. All study participants provided written informed consent.

### Patients

Between October 2013 and May 2015, patients with primary BCS who were scheduled to undergo balloon angioplasty and liver biopsy in the Department of Interventional Radiology in Chinese People's Liberation Army General Hospital were prospectively considered for this study. Inclusion criteria were incomplete obstruction of a hepatic vein or retrohepatic segment of the IVC. These patients were candidates for balloon angioplasty with or without stenting. The exclusion criteria were: liver biopsy specimens with a length < 1.5 cm and/or including < 6 complete portal triads, hepatic space-occupying lesion measuring > 3cm, portal vein thrombosis, congestive heart failure, biliary tract obstruction, primary biliary cirrhosis, and other diseases that affect hepatic sinusoidal pressure.

### Liver histological analysis

Ultrasound-guided percutaneous liver biopsy was performed using 16-gauge Magnum needles (Bard, Covington CA, USA) in the right anterior lower lobe (S5) or right posterior lower lobe (S6) of the liver (according to the Couinaud system of liver segmentation) [26]. The interval between liver biopsy and balloon angioplasty was < 14 days. As stated above, only liver specimens with a length ≥ 1.5cm and including at least six complete portal triads were considered adequate for the study [27].

The specimens were fixed in formaldehyde and embedded in paraffin. The specimens were reviewed using the Metavir scoring system [28] by two pathologists who were blinded to the results and had 12 and 15 years of experiences, respectively. Fibrosis was staged as follows: F0, no fibrosis; F1, portal fibrosis without septa; F2, portal fibrosis and few septa; F3, numerous septa without cirrhosis; and F4, cirrhosis. When there was a divergence of opinions, the higher level of fibrosis stage was adopted.

### SWE

SWE was performed 2 days before angioplasty, and 2 days, 3 months, and 6 months after angioplasty. Two radiologists (H. W. and J. C.) performed the procedures independently. Both two radiologists had at least 7 years of experience in ultrasound examination of the liver and at least 2 months of experience in SWE. They were blinded to the baseline data and biopsy results of the patients.

An ultrasound system (Aixplorer, SuperSonic Imagine, Aix-en-Provence, France) was used for SWE with a convex probe (SC6-1, 1–6 MHz). Liver stiffness was expressed as Young’s modulus according to the formula E = 3ρC^2^, where E is Young’s modulus (kPa), ρ is tissue density (kg/m^3^); and C is shear wave velocity (m/s). The tissue density was assumed to be 1000 kg/m^3^ with the ultrasound system [7].

All patients fasted for at least 8h before SWE. The right arm was placed in maximum abduction to enlarge the space between the ribs. An intercostal space was located in the right anterior lower lobe (S5) or right posterior lower lobe of the liver (S6) (according to the Couinaud system of liver segmentation) and 1.5–3 cm below the liver capsule. A 4cm×3cm SWE box was used to define a 2 cm-diameter circular region of interest (ROI), known as the “Q-box”. The mean, maximal and minimal values of Young’s modulus within the Q- box were measured during a breath hold. Five measurements were performed for each patient with the mean calculated and used for analysis. The Q-box was placed away from the hepatic blood vessels and gall bladder. If the box was not filled completely with color, the measurement was considered a failed measurement, as recommended by the manufacturer.

### Interventional treatment

All patients underwent balloon angioplasty with or without stenting 2 days after satisfactory SWE using the following procedures: modified Seldinger technique was used for puncture of the right femoral vein or the right jugular vein, followed by angiography of the IVC and hepatic vein to locate the site and extent of obstruction. Then the occluded segment(s) were expanded with balloon catheters (Cook Medical, Inc., Winston-Salem NC, USA) to diameters up to 25mm. Hepatic venous pressure (cmH_2_O) was measured during and after angioplasty using a manometer.

Repeat angiography was performed after angioplasty, and unobstructed blood flow was considered a successful angioplasty result. All patients underwent conventional ultrasound examination and SWE at 2 days, 3 months, and 6 months after angioplasty. If a recurrence was suspected, venography was conducted for confirmation.

### Statistical analysis

Statistical analysis was performed with commercial software (IBM SPSS, version 22.0; IBM, Armonk NY, USA). Patient characteristics are reported as mean ± standard deviations. Shapiro–Wilk testing was conducted on all the data. Repeated measures of ANOVA were conducted for liver stiffness at each time point before angioplasty, and 2 days, 3 and 6 months following angioplasty. One-way ANOVA was used to analyze the differences in preprocedure LSMs and pressure values among the different METAVIR fibrosis stages. Least-significant-difference (LSD) post-hoc analysis was employed for pairwise comparison. The LSM of two cases that failed angioplasty before and after surgery was compared using a t-test. The correlation between SWE measurements and venous pressure was analyzed by Pearson correlation. SWE measurements and biopsy results were compared by Spearman correlation. The correlation between preprocedure and postprocedure LSM differences and fibrosis severity was analyzed using the Spearman correlation. P < 0.05 indicated a significant difference.

## Results

### Baseline data

**Fig 1:**
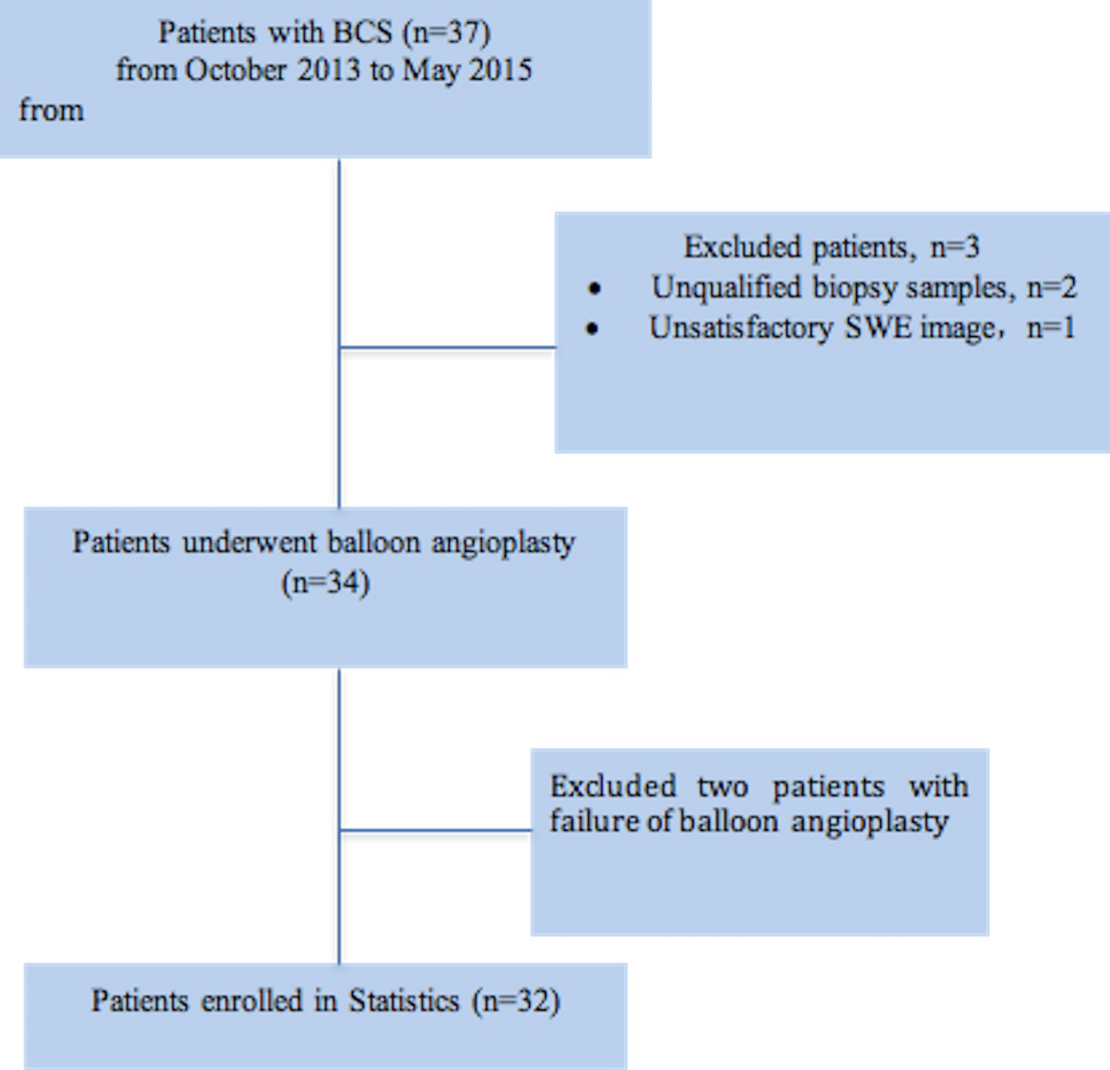
Flowchart of study patients.

Thirty-seven patients were eligible during the recruitment period (Fig 1). Five patients met the exclusion criteria, including two patients with biopsy samples < 1.5 cm long or with < six portal areas under the microscope, one with unsatisfactory SWE images and detection data, and two patients with failure of balloon angioplasty due to length of stenosis. Thus, the final cohort consisted of 32 patients. Shapiro–Wilk testing found that LSM and pressure values conformed to a normal distribution but that fibrosis stages and the elasticity difference before and after angioplasty did not. The patient characteristics are summarized in Table 1.

**Table 1:** Characteristics of the 32 patients.

Characteristics	
Age, mean (SD) [range], years	33.6 (11.1) [15–56]
Male, n (%)	18 (56.2)
BMI, mean (SD) [range], kg/m^2^	21.1(3.2) [14.3–27.3]
Ascites, n (%)	5 (15.6)
BCS obstruction location, n (%)	
Large hepatic vein	6 (18.8%)
Inferior vena cava (IVC)	7 (21.8%)
Combined obstruction of large hepatic veins and IVC	19 (59.4%)
METAVIR fibrosis stage, n (%)	
F0-1	9 (28.1%)
F2	9 (28.1%)
F3	7 (21.8%)
F4	7 (21.8%)

### LSM

**Fig 2:**
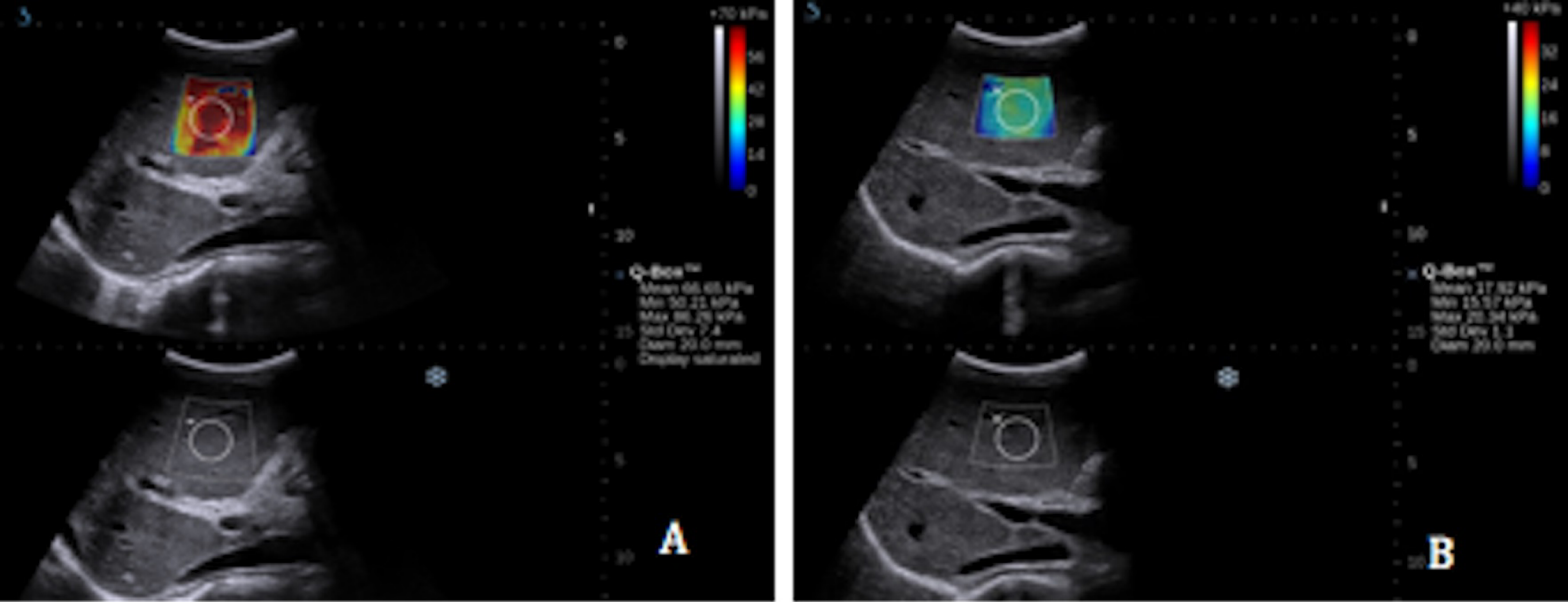
**A.** SWE of one BCS patient 2d before balloon angioplasty, with a mean of 66.65 kPa. **B.** SWE of the same patient 2d after balloon angioplasty, with a mean of 17.92 kPa.

**Fig 3:**
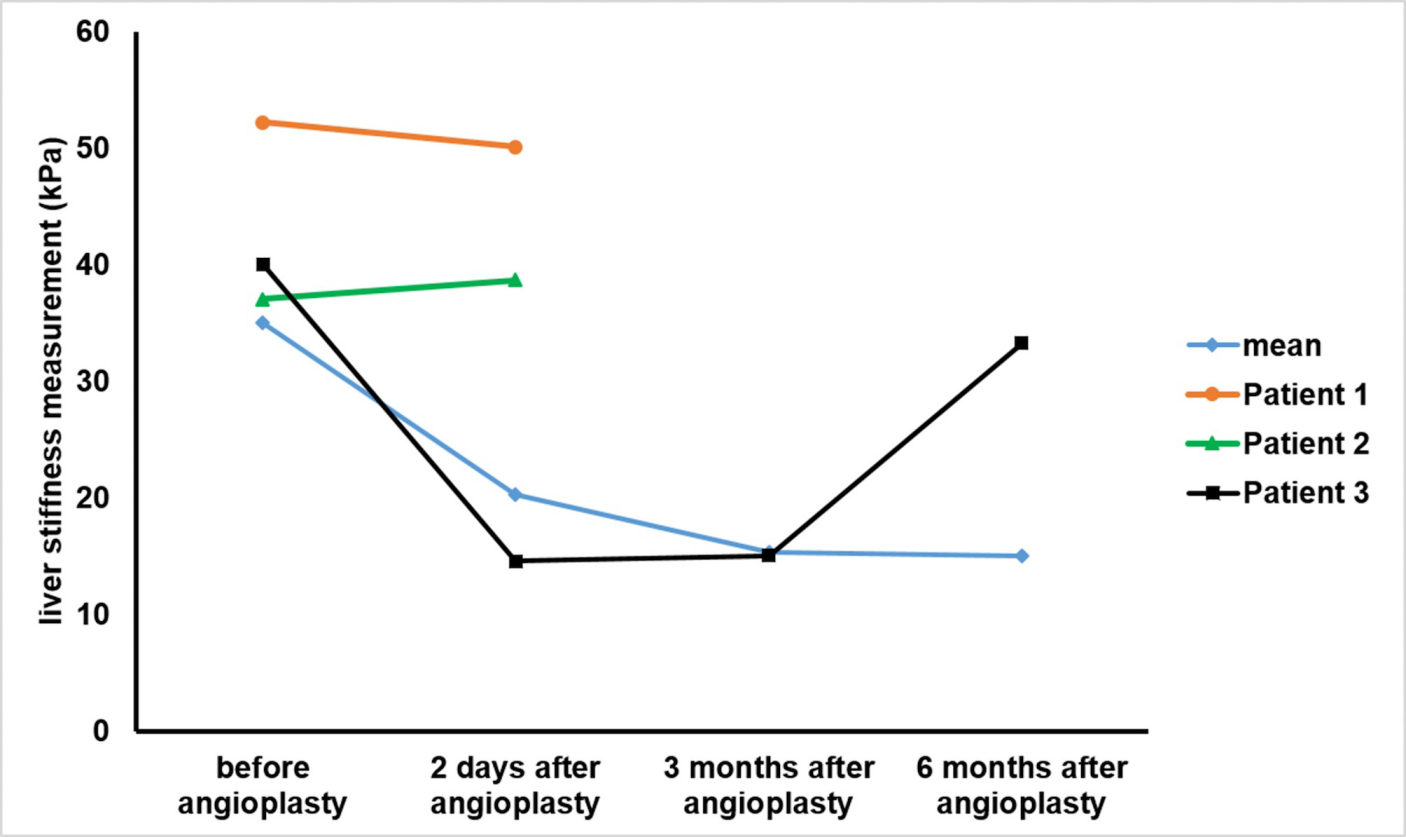
LSM of 34 patients before and after angioplasty (including mean of 32 patients and two failed cases, patient 1 and patient 2, as well as one recurrent case, patient 3).

Thirty-two patients underwent successful balloon angioplasty with or without stenting. LSM after angioplasty decreased significantly (Fig 2A and 2B), with liver stiffness of 35.17 ± 10.60 kPa at 2 d before angioplasty and 20.15 ± 5.47 kPa 2 d after angioplasty (P < 0.001). The mean liver stiffness at 3 months after angioplasty was 15.36 ± 4.34 kPa showing a marked further decrease compared with the measurement at 2d after angioplasty (P < 0.001). The liver stiffness at 6 months after angioplasty was stable at 15.68 ± 5.58 kPa, with no significant differences with the measurement at 3 months after angioplasty (P = 0.636) (Fig 3).

Balloon angioplasty failed in two patients as a result of long-segment stenosis, with LSM of 52.21 kPa, 37.14 kPa before angioplasty and 50.09 kPa, 38.67 kPa after angioplasty, respectively (Fig 3).

Recurrence was detected on angiography in one patient at 6 months after angioplasty. The LSM had been approximately 40.12 kPa at 2d before angioplasty, 14.57 kPa at 2d after angioplasty, 15.14 kPa at 3 months after angioplasty and 33.32 kPa at 6 months after angioplasty. Since the patient was intolerant of warfarin anticoagulation, conservative treatment was administered instead of a second balloon angioplasty.

#### Correlation between LSM and hepatic venous pressure

**Fig 4:**
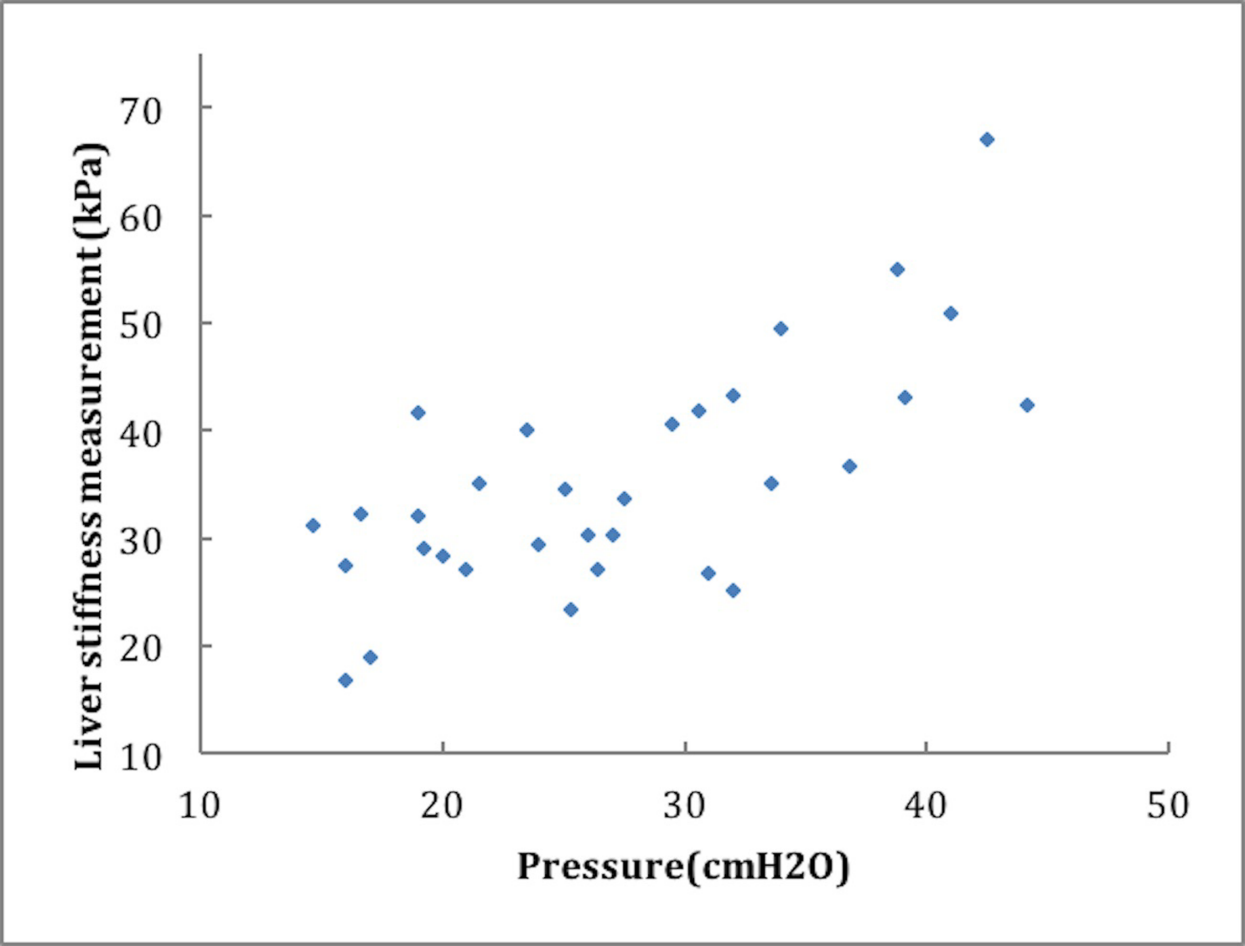
Correlation between LSM and hepatic venous pressure before angioplasty. The correlation coefficient of 32 BCS patients between LSM and hepatic venous pressure before angioplasty was r = 0.701, (P < 0.001).

Mean hepatic venous pressure before angioplasty was 27.18 ± 8.47 cm H_2_O, and that immediately after angioplasty was 16.91 ± 4.48 cm H_2_O (P < 0.001). The coefficient of correlation between LSM and hepatic venous pressure before angioplasty was r = 0.701, (P < 0.001) (Fig 4).

### Correlation between LSM and liver fibrosis

Before angioplasty, LSM did not correlated with the degree of fibrosis in the histologic specimens (r = −0.170, P = 0.22). Two days after surgery, LSM still did not correlated with the degree of fibrosis (r = 0.223, P = 0.220). There was no significant difference in hepatic venous pressures among the different fibrosis stages before angioplasty (F = 1.536, P = 0.227); there was no significant difference in LSM among different fibrosis stages before angioplasty (F = 0.400, P = 0.754). After successful balloon angioplasty, there was no significant difference in hepatic venous pressure among the different fibrosis stages (F = 0.400, P = 0.754); there was no significant difference in LSM among the different fibrosis stages (F = 0.915, P = 0.446). However, LSM difference before and 2 d after angioplasty was negatively correlated with fibrosis (r = −0.502, P = 0.003).

## Discussion

In this study we assessed liver stiffness before and after balloon angioplasty in 32 patients with BCS using SWE. Preprocedure mean LSM was obviously increased above the cut-off value of 10.4 kPa [29, 30] above which is generally considered to reflect F4 fibrosis (liver cirrhosis) in these patients (35.17 ± 10.60 kPa), with one case reaching 67.10 kPa. In patients with congestive heart failure, reversal of cardiac decompensation leading to a significant decrease of the initially increased LSM has been reported [22], but patients’ hepatic venous pressures were not measured, and the relationships between LSM, hepatic venous pressure, and liver fibrosis were not analyzed. We looked at BCS, which causes serious liver congestion, and we found that LSM correlated with hepatic venous pressure (r = 0.701, P < 0.001) rather than with the degree of fibrosis in BCS patients. We speculated that the rise in hepatic sinus tension accompanying congestion and the rise in the viscous elasticity of hepatic tissue resulting in an elevated elastic modulus may explain the increased LSM in BCS patients, but this remains to be investigated.

Because fibrosis does not change over a short period of time, the correlation between liver fibrosis revealed by biopsy and LSM before angioplasty and LSM at 2 d after surgery was investigated. We found LSM did not correlated with fibrosis (r = −0.170, P = 0.22), (r = 0.223, P = 0.220) before and 2 d after angioplasty, respectively. Our findings do not reproduce previous results suggesting that LSM positively correlates with the degree of fibrosis [9, 29]. The reason may be that LSM is more affected by hepatic venous pressure.

Nevertheless, the difference in elasticity 2 d before and 2 d after angioplasty was negatively correlated with the degree of fibrosis (r = −0.502, P = 0.003). Thus the more severe the fibrosis, the smaller the change in liver stiffness after recanalization will be. This is probably because severe fibrosis causes compression and obstruction of the intrahepatic vein and the portal vein still maintains a high pressure even after recanalization. Moreover, at 2 d after recanalization with balloon angioplasty, although the LSM after successful recanalization decreased significantly (20.15 ± 5.47 kPa), it remained far above the cut-off value for liver cirrhosis. One patient showed an obvious increase in liver stiffness during reexamination with SWE 6 months after angioplasty. This patient was diagnosed as having had a recurrence of thrombosis according to the ultrasound. Therefore, SWE may be a method for monitoring the recurrence of BCS patients after angioplasty. Color Doppler ultrasound is less satisfactory in detecting the recurrence of obstruction in BCS patients [31], while LSM can detect it based on its effect on liver stiffness.

The evaluation of the effectiveness of BCS treatment is generally based on symptoms, which inevitably introduces subjective factors and errors [32]. Although Doppler ultrasound has unique advantages as a preferred test for BCS, it has certain technical limitations when used in follow-up examination. Doppler ultrasound may be affected by imaging depth and angle; it has less value in patients with porous and/or membranous lesions of the IVC, and is operator-dependent [31]. Moreover, for lesions with less significant hemodynamic changes, Doppler ultrasound lacks sensitivity in evaluating short- and long-term outcomes after therapy [31]. We discovered that, though unrelated to the degree of liver fibrosis, LSM is highly sensitive to the changes in hepatic venous pressure. Therefore, efficacy of treatment based on SWE can not only improve the outcome evaluation, but also reduce invasiveness.

We are aware of the limitations of our pilot study. First, different in obstruction sites were present among the recruited BCS patients. Despite these differences, liver stiffness was measured uniformly in the right lobe. It was uncertain whether the change in hepatic venous pressure would be uniform for different sites of obstruction. To control for potential differences, LSM was performed at the same site for all patients and IVC was the site of recanalization in most patients (26/32). Among the remaining six patients, four had obvious intrahepatic collateral circulation, so we believe that the site of obstruction was less likely to affect the experimental result. Second, the small sample size is also a limitation. And, there was only one recurrent case. The value of SWE in monitoring BCS recurrence remains to be investigated.

In conclusion, liver stiffness in BCS patients, measured by SWE, decreased considerably after hepatic venous recanalization, and significantly correlated with hepatic venous pressure though not with degree of fibrosis. SWE may be effective in monitoring short- and long-term treatment outcomes of BCS.
